# First study on microscopic and molecular evidences of two bovine hemoplasma species in cattle herds in Al-Qadisiyah Province, Iraq

**DOI:** 10.14202/vetworld.2022.1323-1327

**Published:** 2022-05-26

**Authors:** Yahia Ismail Khudhair, Zeena Fouad Saleh, Hayder N. Ayyez

**Affiliations:** 1 Department of Internal and Preventive Medicine, College of Veterinary Medicine, University of Al-Qadisiyah, Al Diwaniyah, Iraq; 2 Unit of Zoonotic Disease Research, College of Veterinary Medicine, University of Al-Qadisiyah, Al Diwaniyah, Iraq

**Keywords:** hemoplasma spp, microscopic, molecular diagnosis, *Mycoplasma wenyonii*, Iraq

## Abstract

**Background and Aim:**

Hemotropic *Mycoplasmas* are small epierythrocytic bacteria that cause infectious anemia in several livestock species and in humans. Several reports have been made on hemoplasma infections in the south and north of Iraq, but there have been no studies in the middle Euphrates of Iraq. This study aimed to evaluate the presence of hemoplasma species in cattle in Al-Qadisiyah Province, Iraq.

**Materials and Methods:**

Two hundred blood samples were collected from cattle with pale mucous membrane from regions with heavy tick endemicity. The samples were analyzed for the presence of Rickettsia pathogens using thin blood smears and the Diff-Quik stains. All the samples were also examined using polymerase chain reaction (PCR) to amplify the 16S ribosomal ribonucleic acid (rRNA) gene to confirm the presence of the smear-identified microorganisms. Ten PCR positive samples were subjected to 16S rRNA partial gene sequencing to identify the species.

**Results:**

The findings uncovered positivity in 68 (34%) blood smears. PCR revealed positive confirmation in 18 (9%) of the 200 blood samples. *Mycoplasma wenyonii* and *Candidatus mycoplasma hemobos* were identified from 10 PCR positive samples. The nucleotide sequences of the isolates were closely related to isolates from cattle, buffalo, and dogs in Vietnam, Cuba, India, and Germany.

**Conclusion:**

Bovine hemoplasma infections are prevalent in cattle in the Al-Qadisiyah Province in Iraq. Our results may have significance for the development of control programs.

## Introduction

Erythrocytic parasites, also called hemotropic *Mycoplasmas* or hemoplasmas, are part of the newly defined and not well-studied group of Rickettsial pathogens [1,2]. It also includes *Haemobartonella* and *Eperythrozoon* *wenyonii* [3]. They belong to the class Mollicutes in the *Anaplasmataceae* family, according to their morphological features and to *16S* ribosomal ribonucleic acid (*rRNA*) gene sequencing. They are obligatory red blood cell (RBC)-associated pathogens of various mammalian species, including humans [4,5]. In cattle (*Bos taurus*), two distinct hemoplasmas have been identified, *E. wenyonii*, formerly called *Mycoplasma wenyonii* and a provisional species *Candidatus mycoplasma* *hemobos* [6,7]. Distinguishing between these two hemoplasmas is necessary because only *M. wenyonii* has an established etiological significance for mild anemia in cattle [8].

Hemoplasmas are transmitted by blood-feeding arthropods and by direct contact. Hemoplasmas affect animal health alone or concomitantly with other microorganisms [9]. Thermoform, a cattle disease, is characterized by transient fever, anemia, anorexia, depression, edema, lymphadenopathy, decreased milk production, infertility, and reproductive inefficiency [10]. In chronic infection, the disease presents as a subclinical infection with less availability of the microorganisms in peripheral blood samples [11].

Hemoplasma bacteria have a significant impact on livestock outcome as it causes weight loss, reduces milk production, and leads to sudden death [12]. The zoonotic features of the disease cause hematological and neuroprogressive disorders in humans [5].

Information about hemoplasma infections in the middle Euphrates region of Iraq remains scarce and there have been no studies that applied molecular methods so far [13,14]. This study aimed to utilize polymerase chain reaction (PCR) and sequence analysis to determine the presence of hemoplasma species in cattle in the Al-Qadisiyah Province of Iraq and to evaluate genetic associations among local and global strains.

## Materials and Methods

### Ethical approval

The study was approved by University of Al-Qadisiyah, College of Veterinary Medicine (IRAS0822019).

### Study period and location

The study was conducted from January 2019 to December 2020. The animals examined were dairy cattle raised on private dairy farms located in Al Qadisiyah province.

### Samples

Two hundred blood samples (3 mL each) were collected from 200 cattle (age ranging from 6 months to 18 years). Blood was collected from each animal from the jugular vein in ethylenediaminetetraacetic acid-based tubes.

### Direct blood smearing

The blood specimens were examined by a light microscope (Olympus, Japan) with 100× for the presence of bacteria using thin blood smears and Diff-Quik stains (Syrbio, Switzerland). The slides were examined under oil immersion lens (100×) for erythrocyte-associated *Mycoplasma*-like particles.

### PCR

The positive smears were analyzed by PCR based on the *16S rRNA* gene to confirm the presence of the smear-identified microorganisms. Bacterial deoxyribonucleic acid (DNA) was extracted using gSYNC™ DNA Extraction Kit (Geneaid, Taiwan), according to the manufacturer’s protocol. DNA purity and concentration were measured using NanoDrop. The primers were designed using the Primer plus3 program. A 241 bp product of the *16S rRNA* gene was amplified using primers based on the *16S rRNA* gene (Accession No. MT341499.1) in the National Center for Biotechnology Information (NCBI) database. The primers were; Sense primer: 5-ACGAAAGTCTGATGGAGCAAT-3 (219-239) and antisense primer: 5-AGCAAATGCTTTTAACAATG-3 (440-459). The thermocycler program was initial denaturation at 95°C for 10 min, 45 cycles of 95°C for 30 s, 53°C for 30 s, 72°C for 30 s, and final extension at 72°C for 7 min. The PCR products were run through a 1% agar gel and the gel was screened and photographed using an ultraviolet imager.

### *16S rRNA* gene partial sequencing

Purified PCR products from the gels were sent out to Macrogen Company (Korea) for sequencing. The sequencing data were processed using GenBank. The data were analyzed using MEGA X software (Version: MEGA, 11.0.11) to build the phylogenetic tree to find genetic similarities of the study mycoplasmas with global isolates.

## Results

**Figure-1:**
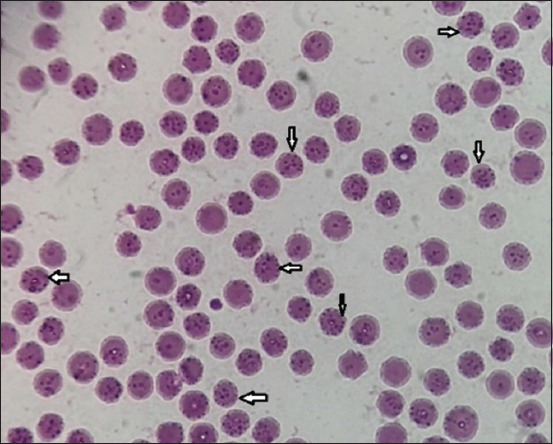
Microscopic appearance of cattle blood smears shows presences of numerous basophilic structures 100×, Diff-Quick stain.

**Figure-2:**
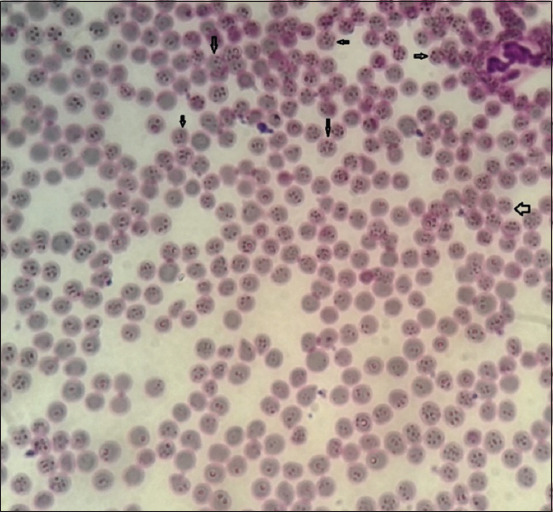
Microscopic appearance of cattle blood smears shows a high load of *Mycoplasma*-like structures present on red blood cells 100×, Diff-Quick stain.

**Figure-3:**
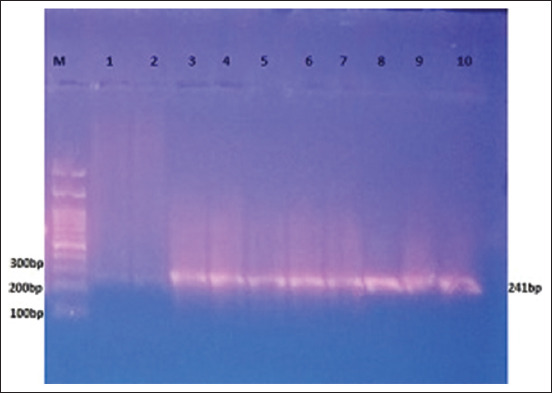
Polymerase chain reaction amplification of *Mycoplasma* spp. in the cattle blood, amplification of the 241 bp fragment of the 16s ribosomal RNA gene (1-10 bands are positive samples, 1 and 2 lines appeared faint bands, it has been considered as positive samples).

A high percentage (34%) of the 68 blood smears had visible hemoplasma-like structures, showing that the RBCs harbored several *Mycoplasma*-like particles (Figures-1 and 2). The PCR revealed the positive confirmation of 18 (9%) out of all utilized samples (Figure-3), 18 (26.5%) out of the 68 positive smear samples, and further some negative blood smears were PCR negative.

**Figure-4:**
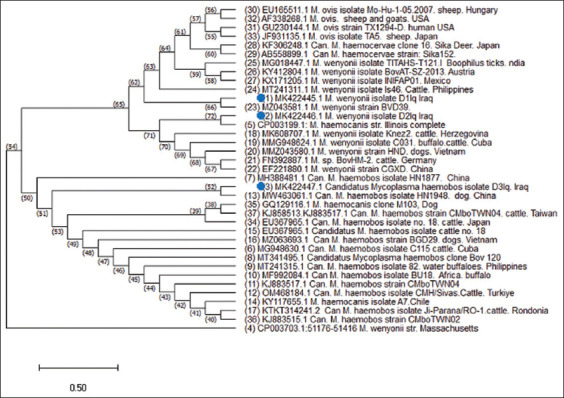
Phylogenetic tree based on the 16S rRNA partial gene sequencing of the polymerase chain reaction products for *Mycoplasma wenyonii* and *Candidatus* *mycoplasma hemobos* (white arrows) detected from cattle in Al-Qadisiyah Province, Iraq.

Ten positive PCR samples were subjected to 16S rRNA partial gene sequencing to identify the *Mycoplasma* species. Two species, namely, *M. wenyonii* and *C. mycoplasma hemobos* were detected. The nucleotide sequences of the isolates were closely similar to isolates from Brazil, Chile, Cuba, and India (Figure-4 and Table-1).

**Table 1:** Estimates of evolutionary divergence between sequences *Mycoplasma wenyonii* and *Mycoplasma hemobos* current isolate distance to other global isolates.*

Mycoplasma spp. strain	MK422445.1	MK422446.1	MK422447.1
MK422445.1 *M. wenyonii* isolate D1Iq. Iraq			
MK422446.1 *M. wenyonii* isolate D2Iq. Iraq	0.02		
MK422447.1 *C.* *mycoplasma* *haemobos* isolate D3Iq .Iraq	0.21	0.21	
CP003199.1: *M. haemocanis* str. Illinois complete genome	2.40	2.60	2.22
CP003703.1:51176-51416 *M. wenyonii* str. Massachusetts	0.01	0.02	0.21
MG948630.1 *C.* *mycoplasma* *haemobos* isolate C115 cattle. Cuba	0.20	0.21	0.01
MH388481.1 *C.* *mycoplasma* *haemobos* isolate HN1877 China	0.19	0.20	0.01
FN392887.1 M. spp. BovHM-2. cattle. Germany	0.20	0.21	0.01
MT241315.1 *C.* *mycoplasma* *haemobos* isolate 82. water buffaloes. Philippines	0.20	0.21	0.01
MF992084.1 *C.* *mycoplasma* *haemobos* isolate BU18. Africa. buffalo	0.20	0.21	0.01
KJ883517.1 *C.* *mycoplasma* *haemobos* strain CMboTWN04. cattle. Taiwan	0.20	0.21	0.01
OM468184.1 *C.* *mycoplasma* *haemobos* isolate CMH/Sivas. Cattle. Turkey	0.20	0.21	0.01
MW463061.1 *C.* *mycoplasma* *haemobos* isolate HN1948. dog. China	0.20	0.21	0.01
FN392887.1 *Mycoplasma* spp. BovHM-2. cattle. Germany	0.20	0.21	0.01
EU367965.1 *C.* *mycoplasma* *haemobos* isolate no. 18. cattle. Japan	0.21	0.21	0.01
MZ063693.1 *C.* *mycoplasma* *haemobos* strain BGD29. dogs. Vietnam	0.21	0.21	0.02
KT314241.2 *C.* *mycoplasma* *haemobos* isolate Ji-Parana/RO-1. cattle. Rondonia	0.20	0.21	0.01
MK608707.1 *M. wenyonii* isolate Knez2. cattle. Herzegovina	0.00	0.01	0.24
MG948624.1 *M. wenyonii* isolate C031. buffalo. cattle. Cuba	0.00	0.01	0.24
MZ043580.1 *M. wenyonii* strain HND. dogs. Vietnam	0.00	0.01	0.24
FN392886.1 *M. wenyonii*. isolate BovHM-8 Cattle. Germany	0.01	0.02	0.24
EF221880.1 *M. wenyonii* strain CGXD. China	0.00	0.02	0.24
MT241311.1 *M. wenyonii* isolate Is46. Cattle. Philippines	0.00	0.02	0.24
MG018447.1 *M. wenyonii* isolate TITAHS-T121.I Boophilus ticks. India	0.01	0.03	0.26
KY412804.1 *M. wenyonii* isolate BovAT-SZ-2013. Austria	0.01	0.03	0.26
KX171205.1 *M. wenyonii* isolate INIFAP01. Mexico	0.01	0.03	0.26
KF306248.1 *C. mycoplasma haemocervae* clone 16. Sika Deer. Japan	0.01	0.03	0.25
AB558899.1 *C. mycoplasma haemocervae* strain: Sika152.	0.01	0.03	0.25
EU165511.1 *M. ovis* isolate Mo-Hu-1-05.2007. sheep. Hungary	0.01	0.03	0.26
GU230144.1 *M. ovis* strain TX1294-D. human. USA	0.02	0.03	0.27
AF338268.1 *M. ovis*. sheep and goats. USA	0.01	0.03	0.26
JF931135.1 *M. ovis* isolate TA5. sheep. Japan	0.01	0.03	0.26
KY117655.1 *M. haemocanis* isolate A7. Chile	0.21	0.22	0.06
KJ858513.1 *M. haemocanis* strain MhcTWN01. dog and cat. Taiwan	0.21	0.22	0.06

*C.* *mycoplasma* *haemobos*=*Candidatus* *mycoplasma* *haemobos*, C. *mycoplasma* *haemocervae*=*Candidatus* *mycoplasma* *haemocervae*, *M. wenyonii*=*Mycoplasma wenyonii*, *M. haemocanis*=*Mycoplasma haemocanis*, *M. ovis=Mycoplasma* *ovis*.

* The number of base substitutions per site from sequences is shown. Analyses were conducted using the maximum composite likelihood model. This analysis involved 30 nucleotide sequences. All ambiguous positions were removed for each sequence pair (pairwise deletion option). There were a total of 266 positions in the final dataset. Evolutionary analyses were conducted in MEGA X. Red color refers to high distance values between enquire nucleotides and database. Green color refers to low distance value between enquire nucleotides and database.

## Discussion

This study was conducted in an area with high tick endemicity. There are about 878 tick species in Iraq [15] that play a significant role in the dissemination of various infectious illnesses in animals and humans [10,16]. Bovine hemotropic *Mycoplasma* widely distributes Rickettsial diseases and their associated risk factors [13]. This study showed that microscopy can be used for the detection of the presence of *Mycoplasma* in blood smear of tick-infested cattle. However, the sensitivity of this method is lower than molecular methods that involve techniques such as PCR and sequencing. The difficulty in diagnosing using blood smears is due to the high latency of these bacteria; in some cases, only a few can be observed in peripheral blood smears [17]. For better diagnosis, molecular tools, such as PCR and sequencing should be used.

Hampel *et al*. [18] reported that the blood smear method has a lower sensitivity (8.3%) for the detection of sheep hemoplasma compared to the gold standard technique, PCR. Nibblett *et al*. [19] detected hemotropic mycoplasma in about 0.9% of blood smears from healthy shelter cats, lower than what was detected using PCR. These cats have a type of subclinical infection that cannot easily be detected using routine peripheral blood smears. Our results showed higher detection of *Mycoplasma* using blood smears, possibly due to the use of the Diff-Quik stain that ensures high-quality visualization of the infectious agent [20]. PCR had a lower detection of the mycoplasmas in the positive smears from affected cattle. This could be due to high nucleotide variability; a mutation could have occurred in a critical binding site that is important for the primer binding to start the PCR amplification process [21,22].

The present study identified isolates highly similar in nucleotide sequences to isolates from Brazil, Chile, Cuba, and India [23]. Our isolates are possibly close to those from these countries because of an evolutionary process that generated new Mycoplasma strains in Iraqi cattle and possibly cattle in neighboring countries such as Saudi Arabia, Jordon, and Lebanon [24].

## Conclusion

*E. wenyonii and C. mycoplasma hemobos* were prevalent in cattle in the Al-Qadisiyah Province of Iraq. In spite ticks hyper endemicity in the study area and high chance of tick-borne pathogens transmis­sion, the parentage of hemoplasma infections were low. The molecular relatedness of Iraqi strains to strains from countries far from Iraq (such as Cuba, Vietnam, China, and Philippines) may have importance for control programs.

## Authors’ Contributions

YIK: Study design, animals’ examination, samples collection, hematological works, and bioinformatics analyses. HNA: Hypothesis, molecular study, and interpretation of results. ZFS: Data collection, literature review, and manuscript preparation. All authors read and approved the final manuscript.
